# Juggling Under Controlled Hypoxia as a Multimodal Coordinative and Cognitive Training in Parkinson’s Disease—A Narrative Review

**DOI:** 10.3390/jfmk11010075

**Published:** 2026-02-12

**Authors:** Dominika Grzybowska-Ganszczyk, Artur Myler, Agata Nowak-Lis, Jarosław Szczygieł, Józef Opara

**Affiliations:** 1 Department of Physiotherapy and Health Sciences, Academy of Physical Education, ul. Mikołowska 72b, 40-065 Katowice, Poland; 48312@awfkatowice.edu.pl (A.M.);; 2 Department of Neurology, Clinical Hospital No.1 Named After Prof. Stanisław Szyszko, ul. 3 Maja 13/15, 41-800 Zabrze, Poland; 3 Humanitas College in Sosnowiec, Humanitas University, ul. Kilińskiego 43, 41-200 Sosnowiec, Poland

**Keywords:** Parkinson’s disease, juggling, cognitive functions, visuomotor coordination, hypoxia, biomarkers

## Abstract

Parkinson’s disease (PD) is a heterogeneous clinical syndrome representing the final stage of a complex and long-lasting neurodegenerative process that involves not only dysfunction of the dopaminergic system but also impairments in other neurotransmitter systems. The diversity of the clinical presentation of PD, together with the existence of Parkinsonian syndromes and atypical Parkinsonism—such as multiple system atrophy (MSA), progressive supranuclear palsy (PSP), and dementia with Lewy bodies (DLB)—has important implications for rehabilitation outcomes and underscores the need for individualized, stage-dependent therapeutic approaches. Juggling is a complex motor activity that integrates cognitive, visuomotor, and balance processes, requiring a high level of concentration, precision, and motor adaptation. In recent years, there has been growing interest in this form of activity as a potential tool for supporting neuroplasticity, cognitive functions, and neurological rehabilitation. The aim of this review was to summarize current scientific evidence on the effects of juggling training on cognitive functions, visuomotor coordination, and balance, as well as to discuss the potential benefits of combining it with controlled hypoxia in patients with Parkinson’s disease (PD). This narrative review additionally considers how disease heterogeneity and stage of progression may influence the effectiveness of such multimodal interventions. This paper reviews the literature concerning the neurophysiological basis of learning to juggle and the mechanisms of brain plasticity, including increases in gray matter volume, improvements in white matter integrity, and reorganization of neuronal networks in motor and associative regions. Attention is drawn to the synergistic potential of combining juggling training with exposure to moderate, controlled hypoxia, which may induce an adaptive response involving the transcription factor HIF-1α, enhance the expression of brain-derived neurotrophic factor (BDNF), and promote angiogenesis and mitochondrial biogenesis. Although juggling and hypoxia are not directly related to training stimuli, both interventions activate overlapping and complementary neuroplastic pathways, providing a conceptual rationale for their parallel consideration and potential integration within future rehabilitation protocols. Juggling delivers task-specific motor–cognitive learning, whereas hypoxia may amplify molecular plasticity signaling, potentially enhancing responsiveness to motor interventions, particularly in patients at early stages of PD when compensatory mechanisms and neuroplastic capacity are relatively preserved. Findings from existing studies suggest that juggling under controlled hypoxic conditions may represent an innovative, safe, and multimodal form of training that supports both cognitive and motor components. Such effects may be particularly relevant in patients at early stages of PD, when compensatory mechanisms and neuroplastic potential are relatively preserved. Such an intervention may contribute to improvements in balance, attention, executive functions, and cognitive flexibility, which is particularly relevant in the context of rehabilitation for patients with neurodegenerative diseases. Importantly, to date, no randomized clinical trials have directly examined juggling performed under controlled hypoxic conditions in PD. Therefore, the present concept should be regarded as translational and exploratory, integrating evidence from juggling-induced neuroplasticity and hypoxia-related physiological adaptations. In this context, the proposed approach represents a proof-of-concept framework for future multimodal interventions rather than an established therapeutic strategy. Available evidence suggests that combining complex sensorimotor skill training with physiological modulation of the internal environment may constitute a novel direction in PD rehabilitation, extending beyond conventional exercise-based models. Despite promising reports, further well-designed clinical studies are needed to determine the optimal training parameters (frequency, intensity, duration, and degree of hypoxia), to evaluate the long-term sustainability of therapeutic effects, and to account for the heterogeneity of PD and related Parkinsonian disorders.

## 1. Introduction

Juggling is a complex motor activity that requires precise integration of cognitive, visuomotor, and balance control functions. During its performance, simultaneous regulation of multiple processes is necessary, including movement planning and programming, trajectory prediction of moving objects, bilateral hand coordination, and rapid sensory information processing. Consequently, juggling represents a unique model of activity engaging both the nervous and musculoskeletal systems, making it an emerging area of interest in neurorehabilitation, movement therapy, and physiotherapy [1]. Research in the field of neuroplasticity confirms that regular engagement in tasks requiring complex motor coordination—such as juggling—leads to lasting structural and functional changes in the brain [2]. Studies have demonstrated, among other findings, an increase in gray matter volume in the parietal, occipital, and temporal cortices, which are responsible for the integration of visual stimuli and motor control [3]. The process of learning to juggle also promotes white matter reorganization, strengthening connections between visual, motor, and cognitive centers [4]. These changes indicate that juggling may serve as an effective tool for stimulating neuroplasticity, which has significant implications for neurological rehabilitation and cognitive therapy [2,5].

The complex nature of juggling engages a broad spectrum of cognitive processes—including selective attention, working memory, predictive ability, and executive control [6]. At the same time, it requires precise visuomotor coordination, interhemispheric cooperation, and dynamic balance regulation. Neuroimaging studies indicate that juggling activates brain regions responsible for motor planning (premotor and supplementary motor cortex), visual stimulus analysis (occipital lobe), and motor error control (cerebellum). In the context of physiotherapy, juggling can be considered a multifactorial exercise that integrates elements of balance, coordination, stimulus response, and motor planning [7]. Its rhythmic character enforces continuous control of the center of gravity, postural stabilization, and adaptation to environmental changes. Regular juggling training has been shown to improve postural stability and balance control through activation of cerebellar and vestibular structures, which may be particularly relevant in therapy for patients with balance disorders resulting from neurological diseases, injuries, or aging processes [8].

Furthermore, juggling positively influences executive functions such as planning, initiation, and behavioral control. Regular practice supports the development of cognitive flexibility, concentration, and divided attention capacity. Therefore, it is applicable not only in motor therapy but also in cognitive interventions for individuals with neurodegenerative diseases and within the elderly population [8,9]. In clinical practice, juggling offers numerous advantages: it is a safe activity that requires neither specialized equipment nor large space, and its intensity can be easily adjusted to the patient’s abilities. The additional element of play and cognitive challenge enhances motivation and engagement in therapy [10].

Despite growing interest, the number of studies addressing the application of juggling in physiotherapy remains limited [7]. To date, the field has been dominated by experimental and pilot reports, often involving small study groups. There remains a lack of conclusive data regarding optimal training parameters (frequency, intensity, duration) and the long-term effects of this form of activity [11]. Therefore, further well-designed studies are needed to assess the effectiveness of juggling across various clinical populations.

In the context of emerging interest in hypoxia-based interventions, it is important to emphasize that not all findings in this field are supported by robust evidence. Yarmush critically evaluated claims suggesting that chronic hypoxia could halt or reverse the progression of Parkinson’s disease, demonstrating that such conclusions were based on a flawed mouse model and an unsafe, clinically non-translatable protocol involving prolonged exposure to 11% oxygen. The author warned that these extreme approaches may generate misleading interpretations driven by nonspecific stress adaptation rather than true neuroprotection. In light of this critique, the concept of safe, moderate, and controlled hypoxia gains particular relevance—especially when combined with coordinative–cognitive tasks such as juggling, which may activate adaptive neuroplastic mechanisms without the risks associated with severe hypoxic exposure [12].

In recent years, increasing attention has been directed toward the use of controlled hypoxia as a therapeutic stimulus in regenerative medicine and neurorehabilitation [13,14,15]. Short-term, moderate hypoxia (intermittent hypoxic training, IHT) activates adaptive mechanisms with neuroprotective, angiogenic, and mitochondrial effects [16,17,18]. A key element of this response is the activation of the transcription factor HIF-1α, which regulates the expression of genes associated with oxygen metabolism, synaptic plasticity, and neuronal survival [19,20]. Studies have shown that moderate hypoxia can increase BDNF levels, support neurogenesis and synaptogenesis, and enhance the functional integrity of neuronal networks [17,19]. Consequently, controlled hypoxia is considered an innovative tool supporting rehabilitation in neurodegenerative diseases such as Parkinson’s disease [17,21].

Recent clinical reports confirm that hypoxic conditioning is a safe method and can be effectively applied in patients with Parkinson’s disease [22]. In a study published in Nature Communications, randomized, controlled, double-blind N-of-1 trials were conducted involving twenty individuals with moderate disease severity. Various continuous and intermittent hypoxia protocols were implemented, with differing oxygen concentrations (FiO_2_ 0.127–0.163). The interventions were well tolerated, and most adverse events were mild and unrelated to the degree of hypoxia. Importantly, intermittent hypoxia at FiO_2_ 0.163 was associated with short-term improvements in motor symptoms reported by participants, although no significant changes were observed in neuroplasticity biomarkers such as BDNF or cortisol. The authors emphasized that these findings provide a starting point for further investigations into the long-term efficacy of this therapeutic approach [22]. These results provide important premises suggesting that the incorporation of controlled hypoxia into multimodal interventions—such as juggling training—may enhance adaptive neuroplastic mechanisms and thereby increase the rehabilitative potential in individuals with Parkinson’s disease [22].

Combining juggling with training under controlled hypoxic conditions may constitute a multimodal rehabilitation intervention in which two mechanisms act synergistically: stimulation of neuroplasticity through complex coordination–cognitive training and adaptive metabolic response induced by hypoxia. Such integration may promote intensified activation of neuronal networks, increased cerebral perfusion, and improved mitochondrial efficiency, potentially leading to enhancement of both motor and cognitive functions.

Neurological disorders remain a leading contributor to global disability. Recent epidemiological analyses indicate that among all neurological conditions, Parkinson’s disease (PD) demonstrates the most rapid rise in prevalence, disability-adjusted burden, and mortality, underscoring its growing public health impact [23].

Within neurodegenerative diseases characterized by progressive neuronal loss—such as dopaminergic degeneration in PD or hippocampal and associative cortical atrophy in Alzheimer’s disease—controlled hypoxia-based interventions have emerged as a potential therapeutic strategy. Experimental evidence suggests that moderate, well-regulated hypoxic exposure may modulate oxidative metabolism, attenuate oxidative stress, and activate endogenous compensatory pathways, including mitochondrial and angiogenic adaptations [8,24,25,26].

Despite these mechanistic insights, clinical research combining hypoxic conditioning with cognitively and motorically demanding tasks—such as juggling—remains scarce. The integration of hypoxia with multimodal training paradigms is theoretically compelling, as it may potentiate neuroplastic responses across multiple functional domains. However, rigorous, adequately powered studies are required to define optimal intervention parameters (e.g., exposure duration, oxygen fraction, training intensity) and to clarify the neurobiological mechanisms that mediate potential therapeutic benefits.

Although juggling and hypoxia are not directly related as training stimuli, both interventions activate overlapping and complementary neuroplastic pathways, which justifies their parallel consideration and the exploration of their potential integration in future rehabilitation protocols. Despite promising preliminary reports, further well-designed clinical studies are required to determine optimal training parameters (frequency, intensity, duration, and degree of hypoxia) and to assess the long-term sustainability of therapeutic effects. At present, the available literature remains limited.

The aim of this article is to provide a review of current evidence regarding juggling as a form of coordination–cognitive training and to present the potential for integrating this activity with controlled hypoxia as a new multimodal rehabilitation concept for neurodegenerative diseases. The paper also outlines directions for future research and the potential clinical applications of this approach.

## 2. Juggling as a Model of Coordination–Cognitive Training

### 2.1. Characteristics of Juggling as a Motor Activity

Juggling is an activity that requires precise planning and real-time movement control, making it one of the most compelling tools for analyzing the interaction between the human nervous system and motor function. During juggling, multiple processes must be synchronized: bimanual coordination, visual control, regulation of muscle tone, and maintenance of body balance [9]. These actions are governed by complex neural networks involving the motor cortex, cerebellum, basal ganglia, and structures responsible for sensory integration.

The process of learning to juggle activates both motor and cognitive systems. It requires mastering movement sequences, predicting object trajectories, maintaining rhythm, and continuously correcting errors. Functional magnetic resonance imaging (fMRI) studies have shown that after only a few weeks of juggling practice, increased activity can be observed in the posterior parietal and occipital cortices—regions responsible for visuo-spatial integration. These observations confirm that juggling is not merely a motor task but also a cognitive one, integrating planning, attention, and spatial control [1].

### 2.2. Neurophysiological Foundations of Juggling

Learning to juggle is strongly associated with neuroplasticity—the capacity of the nervous system to undergo adaptive structural and functional changes [3]. Studies by Draganski et al. demonstrated that after a three-month juggling training period, a significant increase in gray matter volume occurred in the occipital and parietal lobes, reflecting neuronal reorganization processes related to the acquisition of new motor skills [1]. Importantly, after training cessation, these changes partially reversed, suggesting the dynamic nature of brain plasticity.

The cerebellum, a key structure for movement coordination, plays a particularly important role in juggling. It is responsible for comparing intended and actual movement outcomes, enabling precise motor corrections. Additionally, cerebellar–cortical connections support the integration of sensory and motor information, facilitating accurate reproduction of movement trajectories. Cooperation between the cerebellum and vestibular system allows for the maintenance of postural stability under dynamic conditions [27].

Neuronal activation studies have also revealed increased activity in the premotor cortex and anterior cingulate gyrus during juggling, reflecting the engagement of motor planning and error-monitoring processes [28]. Moreover, activity in the temporal lobe indicates the involvement of motion perception and spatial orientation mechanisms. As a result, juggling engages extensive neural networks that connect motor, sensory, and cognitive centers.

These neurophysiological mechanisms are particularly relevant in the context of Parkinson’s disease, where cognitive impairment is highly prevalent and often emerges early in the disease course. Mild cognitive impairment in PD (PD-MCI) affects a substantial proportion of patients and represents the strongest predictor of future dementia. The most commonly affected domains—executive functions, attention, memory, visuospatial processing, and inhibitory control—overlap closely with the cognitive demands of juggling. Because these deficits may precede motor symptoms and progress gradually, interventions that stimulate neuroplasticity across multiple cognitive and motor systems are of particular therapeutic value. Juggling, as a coordinative–cognitive task, directly engages the very domains most vulnerable in PD, making it a promising component of multimodal rehabilitation strategies [29].

### 2.3. Visuomotor Coordination and Postural Balance

Visuomotor coordination constitutes the foundation of effective juggling, enabling precise alignment of motor responses with changing visual stimuli [3]. During performance, the brain must continuously predict the spatial position of objects, analyze their velocity, angle, and flight time, and subsequently generate appropriate motor responses [7]. Studies have shown that jugglers demonstrate superior visuo-spatial processing and shorter reaction times to visual stimuli compared with non-jugglers.

A key role in this process is played by sensorimotor feedback, which allows for real-time correction of motor errors based on visual information. The visual system provides data on object trajectories, whereas the proprioceptive and vestibular systems inform about body position and limb movements. Integration of these inputs occurs primarily in the posterior parietal and occipital cortices, forming a visuo-spatial network [6].

Electroencephalographic (EEG) studies have shown that during juggling, there is an increase in beta rhythm power (15–30 Hz) in motor regions and alpha rhythm in parietal areas, reflecting enhanced neuronal synchronization during visuomotor integration. This phenomenon indicates high processing efficiency under conditions requiring dynamic movement control [3].

Maintaining balance while juggling requires harmonious cooperation among the vestibular, proprioceptive, and visual systems [6]. Even minor shifts in the center of gravity caused by arm and body movements must be compensated by rapid postural reactions. Juggling in a standing position involves constant micro-adjustments of posture, activating trunk and lower limb stabilizing muscles.

Studies have demonstrated that regular juggling training improves parameters of both static and dynamic balance, such as the length of the center-of-pressure (COP) sway path and the velocity of postural oscillations. Neurophysiologically, the maintenance of balance is regulated by the cerebellum, vestibular nuclei, and somatosensory cortex. During juggling, the activity of these structures intensifies, leading to enhanced compensatory capacity of the body. Additionally, juggling stimulates anticipatory postural adjustments, which prepare the muscular system for predicted changes in load and movement [7].

Clinical research has shown that juggling exercises can effectively support the rehabilitation of patients after stroke as well as individuals with vestibular-related balance disorders. Incorporating juggling-based exercises into physiotherapeutic programs promotes improvements in postural stability, muscular reactivity, and overall motor performance.

### 2.4. Cognitive Functions Involved in Juggling

Juggling represents a form of activity that, beyond its motor component, strongly engages cognitive functions—attention, working memory, planning, prediction, and executive control. It requires divided attention, simultaneous monitoring of multiple objects, and the ability to respond to unpredictable environmental changes. Regular juggling practice can lead to improvements in cognitive performance, as evidenced by studies reporting increased cognitive flexibility and concentration following training.

This activity engages the frontoparietal network, responsible for cognitive control and sensory information integration. Increased activity in the prefrontal cortex observed during juggling suggests the involvement of executive supervision and attentional management processes. Concurrently, activation of temporal and occipital regions corresponds to perceptual analysis and visual information integration [3].

Cognitive studies have demonstrated that individuals who practice juggling exhibit better performance in spatial and temporal memory tests and higher efficiency in visual information processing. These mechanisms may result from neuronal adaptations within brain structures responsible for associative learning, prediction, and temporal synchronization. Moreover, learning to juggle may enhance procedural learning processes, similarly to other complex motor activities such as dance or skill-based games [25,28].

## 3. Neuroplastic and Therapeutic Mechanisms of Juggling

### 3.1. Neuroplasticity and Motor Learning

Juggling represents an intriguing form of activity that, due to its combined motor and cognitive structure, can be effectively utilized in physiotherapy. It integrates elements of balance, coordination, rhythm, and attention training, thereby supporting the development of both motor and cognitive abilities across various patient populations. As a complex, multicomponent motor skill (as described above), juggling requires continuous sensorimotor integration and adaptive motor control. Regular juggling practice enhances interhemispheric integration, develops motor planning and postural adaptation skills, and promotes neuronal plasticity. In motor rehabilitation, it may serve as a low-impact yet highly stimulating exercise for both the nervous and muscular systems.

The literature emphasizes that complex motor tasks with cognitive components—such as juggling—can induce neuroplastic changes comparable to those observed in cognitive or musical training. For this reason, juggling finds applications not only in kinesitherapy but also in neurorehabilitation and occupational therapy [6,7,11].

Importantly, evidence from cognitive-training research in Parkinson’s disease indicates that interventions targeting cognition alone produce limited and inconsistent benefits. As demonstrated by Orgeta et al. [30], a recent Cochrane review showed no clear improvement in global cognition, executive functions, visuospatial processing, activities of daily living, or quality of life following 4–8 weeks of cognitive training in individuals with PD-MCI or PDD. Only small, uncertain effects were observed in attention and verbal memory, and the overall certainty of evidence was low due to methodological limitations and small sample sizes. These findings highlight the need for multimodal interventions that simultaneously stimulate motor, sensory, and cognitive systems.

Juggling, by engaging executive control, visuospatial processing, divided attention, and rapid sensorimotor integration, naturally addresses the very cognitive domains most vulnerable in PD. This makes it a promising therapeutic modality capable of eliciting broader neuroplastic adaptations than cognitive training alone [30].

### 3.2. Effects of Juggling on Structural and Functional Brain Changes

In recent years, there has been growing interest in the use of motor activities engaging cognitive functions in the therapy of neurodegenerative diseases. Among these, Parkinson’s disease (PD) represents a key area of research due to its characteristic motor impairments, muscle rigidity, bradykinesia, and balance deficits. In addition to traditional pharmacological and neurosurgical interventions, motor-based therapies with high neurostimulatory potential are increasingly being explored [9,31,32].

A review by McFarthing et al. described a broad spectrum of drugs and strategies under various phases of clinical testing, simultaneously highlighting the limitations of pharmacotherapy as a sole disease-modifying tool [33,34]. Burtscher et al. emphasized the therapeutic potential of hypoxia conditioning as a strategy in Parkinson’s disease, acting through the activation of adaptive cellular pathways and enhancing neuronal resistance to oxidative stress [10,13]. Studies by Jain and colleagues demonstrated that low-oxygen conditions influence mitochondrial metabolism and energy homeostasis, supporting the rationale for experimental hypoxia-based therapies in mitochondrial disorders [15,16]. Ferrari et al. [14] documented that hypoxic therapy may reverse certain neurodegenerative symptoms in animal models, suggesting pro-survival neuronal mechanisms induced by moderate hypoxic stress [35].

A more synthetic overview of hypoxia-related pathways in PD pathogenesis is provided by Gao et al., highlighting the role of HIF-1α and associated neuroprotective mechanisms [21]. More recent studies further suggest that controlled reduction of oxygen delivery under specific experimental conditions may decrease susceptibility to PD or alleviate its progression, with preliminary data from mouse models showing promising outcomes [19].

The application of therapeutic hypoxia was elaborated by Jain et al., who demonstrated that moderate hypoxia improves mitochondrial function and regulates lipid and energy metabolism in neural cells [15,16]. Ferrari et al. [14] confirmed that hypoxic therapy can reverse neurodegenerative symptoms in animal models, enhancing neuronal survival and improving motor performance [14].

Clinical trials have proposed the use of multiple N-of-1 approaches to test therapeutic hypoxia in PD patients, allowing for individualized dosing and monitoring of effects in single subjects [36]. In parallel, other neuroprotective and adjunctive interventions—such as the PD-STAT trial with simvastatin and MARS-PD protocols—are being evaluated, contributing to a broad spectrum of pharmacological and non-pharmacological strategies [37].

Juggling, as a coordination exercise, may be considered a form of non-pharmacological “brain stimulation.” It activates frontoparietal, cerebellar–vestibular, and sensorimotor networks, potentially supporting adaptive mechanisms similar to those induced by controlled hypoxia. Importantly, juggling should not be interpreted as a form of hypoxia per se. Rather, the increased metabolic and oxygen demands associated with complex motor-cognitive activity may create a physiological context in which controlled hypoxic exposure can exert synergistic adaptive effects. In practice, this opens the possibility for synergy between motor (e.g., juggling) and metabolic/environmental (e.g., hypoxia conditioning) interventions within a multimodal rehabilitation framework for patients with PD [15,17,18].

### 3.3. Therapeutic Implications in Neurological Rehabilitation

Neuroplasticity refers to the ability of the nervous system to reorganize its structure and function in response to experience, environmental stimuli, or injury. In physiotherapy, this concept translates into the potential for lasting improvement of motor and cognitive functions through appropriate training. Juggling serves as a model activity that elicits plastic neuronal changes, as it requires continuous learning, error correction, and sensory adaptation [7]. Based on current evidence, the therapeutic potential of juggling-based interventions appears to be greatest in the early stages of Parkinson’s disease, when residual neuroplastic capacity is still relatively preserved and compensatory mechanisms can be effectively engaged.

Neuroimaging studies using MRI, fMRI, and DTI techniques demonstrate that learning to juggle leads to increased gray matter volume in parietal, occipital, and cerebellar regions, as well as strengthened connections among these structures [6]. These changes persist after training cessation, indicating the durable nature of neuronal adaptations. The mechanisms underlying these effects include increased synaptic density, enhanced expression of neurotrophins (BDNF, NGF), and activation of plasticity-related genes such as c-Fos and Arc [11].

In contrast, in more advanced stages of PD—characterized by extensive neurodegeneration, axial symptoms, and reduced cognitive reserve—the effectiveness of complex motor–cognitive training may be limited, necessitating adaptation of task complexity and intensity to individual functional capacity.

During juggling, multiple brain regions are engaged—from visual and motor cortices to subcortical structures and the cerebellum. The complex visuomotor coupling necessitates the integration of sensory information with motor planning, resulting in the activation of cortico-subcortical circuits responsible for attention, working memory, and trajectory prediction [19,20]. These neural processes closely resemble those required for everyday functional tasks, such as walking while performing a simultaneous cognitive task, manipulating objects during household activities, or planning and monitoring goal-directed actions in daily life.

From a physiotherapeutic perspective, juggling is particularly valuable because it develops dual-tasking—the ability to perform two concurrent cognitive–motor tasks. In patients with Parkinson’s disease, this type of training may reduce fall risk, improve balance, and enhance functional independence. Furthermore, juggling introduces rhythmic and sequential components that stimulate dopaminergic activity within the striatum, potentially compensating for dopamine deficiencies characteristic of PD [7]. Clinically, this may translate into improved gait stability during walking with concurrent tasks, better manual dexterity during object manipulation (e.g., carrying groceries, kitchen activities), and enhanced performance of instrumental activities of daily living that require sustained attention and planning.

Studies suggest that even short-term juggling training may improve cognitive flexibility and working memory, supporting the hypothesis of feedback coupling between motor performance and cognitive processes [2,7].

Such improvements are particularly relevant for daily functioning, as they may support attentional control, task switching, and error monitoring in real-world contexts.

An important limitation in implementing early, targeted rehabilitation strategies in Parkinson’s disease is the difficulty of accurate diagnosis in the prodromal and early clinical stages. Clinical diagnostic criteria remain partially nonspecific, and early symptoms may overlap with atypical Parkinsonian syndromes or other neurological conditions.

Emerging evidence highlights the growing role of immunological and inflammatory markers in differentiating PD from other neurodegenerative disorders; however, these approaches are still under investigation and lack sufficient specificity for routine clinical use.

Positron emission tomography (PET), particularly with dopaminergic tracers, remains one of the most reliable tools for identifying presynaptic dopaminergic dysfunction and may be considered functionally patognomonic in ambiguous cases. Nevertheless, its high cost, limited availability, and restricted access significantly constrain its widespread clinical application, especially in early-stage or screening contexts. These diagnostic challenges further underscore the importance of individualized, flexible rehabilitation approaches that can be adapted to heterogeneous clinical presentations and varying degrees of diagnostic certainty.

## 4. Training Under Controlled Hypoxic Conditions

### 4.1. Physiological Foundations of Adaptation to Hypoxia

Both juggling and therapeutic hypoxia rely on stimulating the natural adaptive mechanisms of the nervous system. In both cases, activation of the cellular stress axis, increased mitochondrial activity, and enhanced expression of trophic factors are observed. During juggling practice, a similar induction of BDNF and improved cerebral blood flow in motor and visual cortical areas have been reported. Both forms of stimulation also contribute to an increase in white matter connectivity and synchronization of interhemispheric neuronal activity [19].

Therapeutic hypoxia, as discussed in this review, refers specifically to normobaric hypoxia, defined as a controlled reduction in the fraction of inspired oxygen (FiO_2_) while maintaining normal atmospheric pressure. Unlike hypobaric hypoxia experienced at high altitude, normobaric hypoxia allows precise regulation of oxygen availability under safe and reproducible laboratory or clinical conditions [13].

Normobaric hypoxia is typically delivered using hypoxicators or gas-mixing systems connected to face masks or breathing hoods, which provide a hypoxic air mixture with reduced FiO_2_ while allowing spontaneous, continuous breathing. Importantly, therapeutic hypoxia does not involve breath holding, intermittent apnea, or voluntary respiratory restriction. Participants breathe normally throughout the intervention, with ventilation adapting naturally to the reduced oxygen content of the inspired air.

In clinical and experimental studies, normobaric hypoxia protocols most commonly use FiO_2_ values ranging from approximately 12% to 15%, corresponding to simulated altitudes of ~2500–4000 m above sea level. Exposure duration typically ranges from 20 to 60 min per session, with frequencies of 2–5 sessions per week over several weeks, depending on the study population and therapeutic goal.

At the cellular level, moderate hypoxic exposure activates hypoxia-inducible factor-1α (HIF-1α), leading to upregulation of genes involved in angiogenesis, glucose metabolism, mitochondrial biogenesis, and neuroprotection. These mechanisms include increased expression of vascular endothelial growth factor (VEGF), erythropoietin (EPO), and neurotrophic factors such as BDNF, all of which are relevant to neuronal survival and plasticity [13].

From a neurophysiological perspective, juggling may therefore be regarded as a form of increased metabolic and cognitive demand rather than hypoxia per se, in which elevated neural activation and energy requirements create conditions that may potentiate neuroplastic adaptations. Accordingly, juggling should not be equated with hypoxia itself, but rather considered a cognitively and metabolically demanding stimulus that may act synergistically with controlled hypoxic exposure. Combining the two methods—controlled hypoxia and juggling—may, in the future, form the basis of a novel multimodal rehabilitation concept for neurodegenerative disorders, particularly Parkinson’s and Alzheimer’s diseases [19,20]. Such a combined approach may allow simultaneous engagement of motor, cognitive, and metabolic adaptive pathways, while maintaining a high level of safety and individualization.

### 4.2. The Role of HIF-1α and Neuroprotective Mechanisms

The concept of integrating cognitively demanding coordination exercises, such as juggling, with protocols of moderate hypoxic exposure is based on the assumption that both interventions activate complementary adaptive mechanisms. These include modulation of mitochondrial metabolism, induction of neurotrophic factors (including BDNF and NGF), and activation of cellular defense pathways dependent on the transcription factor HIF-1α (hypoxia-inducible factor 1-alpha).

Under conditions of moderate hypoxia, HIF-1α becomes stabilized; under normoxic conditions, it is rapidly degraded through hydroxylation and proteasomal breakdown. When stabilized, HIF-1α binds to its subunit HIF-1β, forming a complex that regulates the expression of numerous genes responsible for adaptation to reduced oxygen availability. The most important of these are those encoding erythropoietin (EPO), vascular endothelial growth factor (VEGF), and glycolytic enzymes that increase the efficiency of energy production under low partial oxygen pressure [19].

In the nervous system, HIF-1α plays a crucial role in maintaining neuronal energy homeostasis, stimulating angiogenesis and improving cerebral perfusion, while also indirectly enhancing the expression of brain-derived neurotrophic factor (BDNF)—a protein responsible for synaptic plasticity, learning, and memory processes [20]. Additionally, activation of HIF-1α triggers antioxidative and anti-inflammatory mechanisms, including increased expression of heme oxygenase-1 (HO-1) and enzymes responsible for detoxifying reactive oxygen species. Consequently, moderate hypoxia can exert a neuroprotective effect by limiting oxidative stress and neuronal apoptosis [22].

From the standpoint of neurological rehabilitation, combining such an adaptive response with a cognitively demanding motor activity (e.g., juggling) may enhance neuroplastic processes through simultaneous stimulation of metabolic pathways and neuronal networks. Theoretically, this could lead to improved mitochondrial efficiency, enhanced cerebral blood flow, and better cognitive and motor performance [14,18].

In clinical practice, however, this approach requires rigorous safety evaluation and standardization of hypoxic exposure parameters—including duration, degree of oxygen reduction, and exercise intensity. Current data on controlled hypoxia training are predominantly experimental and preclinical, and most studies combining hypoxia with physical activity (including juggling) involve small pilot samples [19,20]. Therefore, future research should include randomized clinical trials with detailed monitoring of cardiometabolic and neurological parameters to confirm the safety and efficacy of such a multimodal intervention.

## 5. Rationale for Combining Juggling Training with Controlled Hypoxia

Although juggling itself does not induce systemic hypoxia, both juggling and controlled hypoxic exposure activate partially overlapping neuroplastic mechanisms, including increased expression of brain-derived neurotrophic factor (BDNF), angiogenesis, mitochondrial biogenesis, and reorganization of motor–associative neuronal networks. Juggling primarily provides task-specific motor–cognitive learning, whereas hypoxia acts as a physiological modulator that amplifies molecular plasticity signaling via HIF-1α–dependent pathways. 

Although no studies have directly examined juggling performed under hypoxic conditions, converging evidence from juggling-induced neuroplasticity and hypoxia-augmented motor or cognitive training suggests the potential for synergistic effects. Both interventions independently engage shared biological pathways, including enhanced BDNF signaling, angiogenesis, mitochondrial biogenesis, and network reorganization within motor–associative circuits. These common mechanisms provide the conceptual foundation for a potential synergistic interaction. Accordingly, the present review adopts a translational framework integrating these two lines of evidence.

### 5.1. Studies Combining Juggling and Hypoxia

To date, no randomized controlled trials have investigated juggling performed under conditions of controlled hypoxia. This complete absence of direct empirical evidence highlights a substantial research gap and underscores the exploratory and hypothesis-generating nature of the proposed integrative approach.

### 5.2. Hypoxia-Augmented Motor and Cognitive Rehabilitation

Given the lack of studies directly combining juggling with hypoxia, this section summarizes available evidence from investigations integrating hypoxic exposure with motor or cognitive training to establish a translational model linking juggling-induced neuroplasticity with hypoxia-enhanced training paradigms.

Only a limited number of studies have examined hypoxic training in neurological or cognitively impaired populations. Available data suggest that moderate, controlled hypoxia combined with motor or cognitive tasks may improve executive functions, working memory, and overall functional performance, potentially mediated by HIF-1α–dependent pathways, increased BDNF expression, angiogenesis, and mitochondrial biogenesis (Table 1). These findings support the concept that hypoxia may serve as a physiological enhancer of training-induced neuroplasticity rather than as a stand-alone therapeutic stimulus.

Importantly, physical training under controlled hypoxia has also been investigated in non-neurological clinical populations, including cardiac rehabilitation. Our previous narrative review demonstrated the feasibility of hypoxic exposure and its potential cognitive benefits in patients with cardiovascular diseases, highlighting hypoxia-dependent modulation of cognitive function and neuroplastic mechanisms. However, to our knowledge, no comparable studies exist in Parkinson’s disease populations.

Collectively, existing evidence suggests that combining task-specific motor–cognitive training with controlled hypoxia represents a promising translational strategy that warrants further investigation in neurodegenerative conditions.

### 5.3. Juggling and Neuroplasticity: Evidence Base

Numerous neuroimaging and behavioral studies have demonstrated that juggling training leads to increases in gray matter volume, improvements in white matter integrity, and reorganization of neuronal networks within motor and associative regions (Table 2). These effects have been observed in both younger and older adults. Although these interventions show cognitive and motor benefits, none have been conducted in Parkinson’s disease populations, and none have incorporated hypoxic exposure.

## 6. Multimodal Neuroplastic Stimulation Through Juggling and Hypoxia

The results of existing studies indicate that juggling is one of the few forms of physical activity that effectively integrates motor, cognitive, and emotional components. It constitutes a complex neuromotor stimulus capable of inducing plastic changes in both cortical and subcortical structures, particularly within the parietal and occipital lobes, the cerebellum, and the corpus callosum. Observed modifications include increased gray matter density and improved white matter integrity, reflecting functional enhancement of perceptual-motor processing.

Learning to juggle activates complex neuronal networks that engage not only motor centers but also regions responsible for attention, working memory, and motor planning. This process is inherently interactive—each cycle of error and correction promotes synaptic reorganization and strengthens neuronal pathways. Repetitive juggling practice has been shown to increase levels of brain-derived neurotrophic factor (BDNF), promoting neurogenesis and angiogenesis within the motor cortex and hippocampus.

From a neurophysiological perspective, juggling can be viewed as a form of adaptive neuronal stress which—similarly to moderate hypoxia—stimulates the nervous system’s adaptive mechanisms through HIF-1α and BDNF pathways. As a result, it enhances neuronal oxygen metabolism, strengthens antioxidant defense, and activates neuroprotective processes [42].

### 6.1. Practical Applications of Juggling in Physiotherapeutic and Neurological Rehabilitation

From a physiotherapeutic perspective, juggling offers several advantages that make it an attractive component of motor therapy. First, it is a low-injury-risk exercise requiring minimal equipment. Second, by engaging cognitive (attention, planning, trajectory prediction) and sensory functions (visuomotor coordination, proprioception), juggling facilitates sensory integration in ways that traditional balance training often does not [43].

In neurological therapy, juggling may serve as a coordination-cognitive training modality. In Parkinson’s disease—where dopaminergic communication and executive functions are impaired—regular juggling practice may support movement rhythmization, improve kinetic fluidity, and reduce bradykinesia. Combining this activity with methods that enhance neuroplasticity—such as controlled hypoxic exposure or interval training—could potentially increase rehabilitation efficacy [44].

Moreover, juggling can be easily scaled, ranging from simple one- or two-ball exercises to complex multi-object or whole-body movement patterns. This flexibility allows the therapist to individually adjust task difficulty and gradually increase cognitive load. Importantly, the playful and engaging character of juggling enhances patient motivation and satisfaction—factors crucial for maintaining long-term therapy adherence [45].

### 6.2. Shared Neuroplastic Mechanisms of Juggling and Hypoxia

Both interventions activate overlapping biological pathways, including

- Enhanced BDNF signaling;
- Increased angiogenesis;
- Mitochondrial biogenesis;
- Elevated neuronal metabolic activity;
- Reorganization of motor networks;
- Activation of HIF-1α (specific to hypoxia).

These shared mechanisms provide a biological rationale for combining task-specific motor learning with hypoxia-mediated amplification of plasticity signaling.

### 6.3. Perspectives for the Development of Multimodal Rehabilitation Programs

Despite promising findings, juggling as a therapeutic tool remains relatively underexplored compared to other physical activities used in neurological rehabilitation. Existing reports primarily focus on short-term training effects, often in studies with limited sample sizes. Future research should therefore include randomized clinical trials employing advanced neuroimaging techniques—such as magnetic resonance imaging (MRI), diffusion tensor imaging (DTI), and functional near-infrared spectroscopy (fNIRS)—to determine the localization, extent, and dynamics of plastic changes induced by juggling, and to identify which neural regions are responsible for observed improvements in cognitive and motor functions.

Another important research direction involves assessing the durability of juggling’s effects—its influence on long-term retention of cognitive functions, postural control, and the ability to transfer learned skills to activities of daily living (ADL). It is also valuable to examine how juggling training affects quality of life, independence, and psychosocial outcomes in patients with various neurological conditions, such as Parkinson’s disease or mild cognitive impairment.

In the context of integration with hypoxic therapy, defining optimal exposure parameters—duration, oxygen reduction level, session frequency, and exercise intensity—will be critical for maximizing adaptive effects while maintaining safety. Further studies should explore tolerance and physiological effects of such interventions in neurological patients, especially in those with coexisting cardiovascular or metabolic disorders.

A significant direction in the development of multimodal rehabilitation programs involves the integration of digital technologies. The use of virtual reality (VR), augmented reality (AR), and motion capture systems allows for precise patient progress monitoring, task personalization, and increased engagement through gamification elements. In the future, these technologies may support physiotherapists in adjusting training loads, analyzing movement quality, and documenting therapeutic outcomes in real time.

Juggling, with its high neuroplastic potential and low injury risk, represents a promising form of complex coordination-cognitive training. Its inclusion in rehabilitation programs may enhance cognitive and motor functions as well as balance in patients with diverse neurological disorders. Combining this activity with innovative metabolic stimulation techniques, such as controlled hypoxia, opens new perspectives for rehabilitation grounded in the adaptive mechanisms of the nervous system.

Future research should focus on standardizing therapeutic protocols, verifying their clinical efficacy across patient populations with varying degrees of deficit, and developing modern tools to support physiotherapists in practical implementation. The long-term goal is to create multimodal, personalized rehabilitation programs that optimally integrate cognitive, motor, and metabolic stimulation to support neuroregeneration and improve patient quality of life.

## 7. Summary and Conclusions

### 7.1. General Conclusions

1. Juggling is a complex coordination task that naturally activates multiple brain regions responsible for motor control, attention, working memory, and motor planning.
2. Regular juggling practice leads to measurable neuroplastic changes, including increased gray matter volume and improved white matter integrity in brain structures associated with motor learning.
3. This activity benefits not only motor abilities but also cognitive functions—enhancing attention, memory, information-processing speed, and visuomotor coordination.
4. The adaptive mechanisms underlying juggling share partial similarities with processes observed in response to moderate hypoxia, involving activation of HIF-1α pathways and increased BDNF expression.
5. There is scientific evidence suggesting that combining juggling with controlled hypoxia or other forms of metabolic training may further enhance neuroplastic processes and improve the efficacy of neurological therapy.
6. Importantly, the potential effectiveness of juggling-based and hypoxia-supported interventions appears to be strongly dependent on the stage of Parkinson’s disease, with the greatest benefits expected in early stages characterized by preserved neuroplastic capacity.
7. In more advanced stages of PD, extensive neurodegeneration, axial symptoms, and cognitive decline may limit responsiveness to complex motor–cognitive interventions, necessitating individualized adaptation or alternative rehabilitation strategies.

### 7.2. Practical Implications for Physiotherapists

1. Juggling can be integrated as a supportive component in the therapy of neurological, orthopedic, and geriatric patients—particularly when the goal is to improve balance, coordination, and cognitive function.
2. Juggling training should be introduced gradually, beginning with simple one- or two-ball exercises, increasing the number of objects only after stable motor control is achieved.
3. To elicit meaningful neuroplastic effects, training frequency should be at least three times per week for 15–20 min per session.
4. In Parkinson’s disease, juggling can be used as a form of rhythmic motor training that enhances gait fluidity and postural control.
5. In patients after stroke or with mild cognitive impairment, juggling exercises may support sensorimotor integration and improve divided attention.
6. For older adults, juggling represents a safe and engaging method to maintain motor fitness and prevent falls.
7. Combining juggling with other training modalities—such as balance, rhythmic, or hypoxic exercises—may produce synergistic effects in promoting neuronal plasticity.
8. Physiotherapists should monitor patient progress and adjust task difficulty according to psychomotor capacity and functional safety.
9. In patients with Parkinson’s disease, careful patient selection based on disease stage, cognitive status, and postural stability is essential to ensure both safety and therapeutic effectiveness.
10. Appropriate clinical diagnosis and differentiation from atypical Parkinsonian syndromes should precede the implementation of complex multimodal rehabilitation programs.

### 7.3. Recommendations for Future Research

1. Randomized controlled trials should be conducted to evaluate the effects of juggling on cognitive function, balance, and neuroplasticity across different patient populations.
2. Neuroimaging techniques (MRI, DTI, fNIRS) should be employed to monitor structural and interhemispheric connectivity changes resulting from juggling training.
3. It is necessary to determine the optimal training parameters—frequency, duration, and intensity—for various clinical populations.
4. Future studies should investigate the combined effects of juggling and hypoxic therapy on neuronal metabolism and functional recovery.
5. Long-term studies are needed to assess the durability of juggling-related cognitive and motor improvements and their impact on patients’ quality of life.
6. The use of digital technologies—such as VR systems, motion sensors, and mobile applications—should be explored to personalize and monitor juggling training in both clinical and home settings.
7. Development of interdisciplinary therapeutic protocols combining physiotherapy, neuropsychology, and neurobiology is recommended to fully exploit the neuroplastic potential of this activity.
8. Future research should explicitly account for the clinical and biological heterogeneity of Parkinson’s disease, including disease stage, symptom profile, and diagnostic certainty, when evaluating the efficacy of juggling- and hypoxia-based interventions.

## 8. Novelty and Translational Perspective

This review is the first to propose a conceptual model integrating juggling-based motor–cognitive training with controlled hypoxia as a potential multimodal rehabilitation strategy for Parkinson’s disease. Despite encouraging indirect evidence, further well-designed clinical trials are required to determine optimal intervention parameters (frequency, intensity, duration, and degree of hypoxia) and to evaluate the long-term sustainability of therapeutic outcomes.

## Figures and Tables

**Table 1:** Studies combining motor or cognitive training with hypoxic exposure.

Author	N	Population	Motor/Cognitive Intervention	Hypoxia Protocol	Main Outcomes	Limitations
Burtscher et al. [13]	34	Healthy older adults	Coordination training	Normobaric hypoxia (FiOâ‚‚ 14%)	Improved executive functions	Small sample size
Schega et al. [38]	28	Mild cognitive impairment (MCI)	Cycling + cognitive tasks	Simulated altitude ~3000 m	Improved working memory	No follow-up
Grzybowska-Ganszczyk et al. [39]	-	Cardiac patients MCI	Narrative review of hypoxia + cognitive effects	-	Hypoxia interacts with cognitive decline mechanisms; possible benefit of integrated hypoxic training	Narrative review (no intervention outcomes)
Nowak-Lis et al. [40]	36	Patients after myocardial infarction	Endurance rehabilitation training	Normobaric hypoxia (~3000 m) vs. normoxia	Hypoxic training improved spiroergometric parameters, exercise tolerance, and hemodynamic measures	Focus on cardiac rehab; not neurological/cognitive outcomes

**Table 2:** Key studies on juggling-induced neuroplasticity and cognitive effects.

Author (Year)	N	Population	Intervention Duration	Main Effects	Method
Draganski et al. [1]	24	Young adults	3 months of juggling	Increased gray matter in hMT/V5	VBM MRI
Boyke et al. [9]	44	Older adults	3 months of juggling	Increased gray matter in hMT/V5 and associated regions	VBM MRI
Scholz et al. [4]	48	Healthy adults	6 weeks of juggling	Increased fractional anisotropy in white matter	DTI MRI
Voelcker-Rehage & Willimczik [41]	1206	Broad age range (6-89 years)	6 juggling sessions	Improved motor performance	Behavioral

## Data Availability

The raw data supporting the conclusions of this article will be made available by the authors on request.
